# Electrically Driven Liquid Crystal Elastomer Self-Oscillators via Rheostat Feedback Mechanism

**DOI:** 10.3390/polym17050617

**Published:** 2025-02-25

**Authors:** Kai Li, Zuhao Li, Lin Zhou

**Affiliations:** 1 School of Civil Engineering, Anhui Jianzhu University, Hefei 230601, China; kli@ahjzu.edu.cn (K.L.); zuhaoli@stu.ahjzu.edu.cn (Z.L.); 2 School of Mechanical and Electrical Engineering, Anhui Jianzhu University, Hefei 230601, China

**Keywords:** rheostat feedback mechanism, liquid crystal elastomer, self-oscillation, multi-scale method, bifurcation analysis

## Abstract

The reliance of feedback mechanisms in conventional light-fueled self-oscillating systems on spatially distributed light and intricately designed structures impedes their application and development in micro-robots, miniature actuators, and other small-scale devices. This paper presents a straightforward rheostat feedback mechanism to create an electrically driven liquid crystal elastomer (LCE) self-oscillator which comprises an LCE fiber, a rheostat, a spring, and a mass. Based on the electrothermally responsive LCE model, we first derive the governing equation for the system’s dynamics and subsequently formulate the asymptotic equation. Numerical calculations reveal two motion phases, i.e., static and self-oscillating, and elucidate the mechanism behind self-oscillation. By employing the multi-scale method, we identify the Hopf bifurcation and establish the analytical solutions for amplitude and frequency. The influence of various system parameters on the amplitude and frequency of self-oscillation was analyzed, with numerical solutions being validated against analytical results to ensure consistency. The proposed rheostat feedback mechanism can be extended to cases with rheostats that have more general resistance properties and offers advantages such as simple design, adjustable dimensions, and rapid operation. The findings are expected to inspire broader design concepts for applications in soft robotics, sensors, and adaptive structures.

## 1. Introduction

A self-oscillating system autonomously generates periodic motion in response to steady stimuli, without requiring external periodic driving forces [1,2]. The advantages of self-oscillation include simpler system design, improved energy efficiency, and adaptability, enabling the system to modify its oscillatory behavior based on environmental variations [3,4]. Furthermore, self-oscillating systems typically demonstrate significant mechanical robustness when subjected to specific disturbances [5]. Self-oscillation phenomena are widely present in mechanical engineering [6,7], chemistry equipment [8], and energy harvesters [9], holding significant application value in these fields. Understanding and leveraging self-oscillation phenomena can lead to innovations and optimizations across various domains, offering extensive practical applications and research prospects.

The diversity of active materials provides abundant possibilities for constructing self-oscillating systems. Commonly observed active materials include hydrogels [10,11,12], ion gels [13], dielectric elastomers [14], thermally responsive polymers [15], and liquid crystal elastomers (LCEs) [16,17,18,19,20]. Various self-oscillating modes are suggested to balance energy dissipation in motion through the input of net energy [21,22,23,24,25,26]. Chaos [27,28], jumping [29,30,31], rotation [32,33,34,35], swinging [36,37,38], bending [39], buckling [40,41], vibration [42,43], rolling [44], twisting [45], and swimming [46] are all examples of these self-oscillating modes. Even the synchronized self-oscillating of coupled liquid crystal oscillators can be observed [47,48]. To further illustrate these modes, specific mechanisms demonstrate their functioning. For example, the repetitive self-shadowing effect mechanism enables local rotational or displacement self-oscillation [49,50]. The large deformation coupling mechanism exhibits self-oscillation behaviors in materials [51,52]. The mechanism of photothermal surface tension gradient can trigger complex self-oscillation phenomena at the material interface [53,54,55,56,57].

The intelligent responsive materials mentioned above have attracted attention due to their efficient energy conversion, pollution-free nature, and strong tunability [13,58,59,60,61,62]. LCEs, as advanced intelligent materials, exhibit anisotropy and bidirectional shape memory properties [63], enabling the internal structure to flexibly transition between monodomain and polydomain states in response to external stimuli such as light [16], heat [64], and electric [65] or magnetic fields [66]. The transition induces macroscopic deformation, resulting in diverse forms and structural behaviors [67,68,69,70,71]. Leveraging specific characteristics of LCE materials, including robustness, light weight, and contactless response, numerous LCE self-oscillating systems have been realized through various feedback mechanisms, which has significant applications in artificial intelligence [72], optical components [73], and sensors [74].

Currently, the reliance of feedback mechanisms in conventional light-fueled self-oscillating systems on spatially distributed light and intricately designed structures impedes their application and development in micro-robots, miniature actuators, and other small-scale devices. Previous research has demonstrated the potential of electrically driven LCE actuators in various applications, such as the electric bending actuation of ionic liquid crystal elastomers and ferroelectric liquid–crystalline elastomer films, laying the foundation for innovative designs [75,76]. By utilizing the deformation of the LCE to couple the movement of the rheostat slider for current adjustment, this paper presents a straightforward rheostat feedback mechanism to create an electrically driven LCE self-oscillator which comprises an LCE fiber, a rheostat, a spring, and a mass. We aim to establish an electrothermally responsive LCE model to derive the dynamic behavior of the LCE self-oscillator, explain the self-oscillation mechanism, and analyze how system parameters affect its amplitude and frequency. The proposed rheostat feedback mechanism can be adapted for rheostats with more versatile resistance properties, offering advantages like simplified design, adjustable dimensions, and quick response. These findings are anticipated to inspire innovative design concepts for applications in soft robotics [77], adaptive structures [78], and energy harvesting [79].

This paper is structured into the following sections: Section 2 establishes the dynamic model of the electrically driven LCE self-oscillator and conducts force analysis to derive the governing equation, introducing the model of the electrothermally responsive LCE. In Section 3, we evolve the governing equation into an asymptotic equation for the electrically driven LCE self-oscillator. Section 4 examines two motion phases, i.e., static and self-oscillating, by using both governing and asymptotic equations. In Section 5, we employ the Hurwitz criterion to analyze the system’s bifurcation points and then obtain the amplitude and frequency formulas via the multi-scale method. Section 6 analyzes the impact of various parameters on the triggering conditions, amplitude, and frequency of self-oscillation. Finally, Section 7 presents the conclusions.

## 2. Theoretical Model and Formulation

A recent study by Xu et al. presents a self-sustained soft robot that achieves continuous movement by using electrically driven LCEs. The innovation integrates LCEs with liquid metal (LM) to create an actuator that enables continuous motion without the need for periodic power switching. This experimental work provides valuable insights into the practical realization of electrothermally responsive LCE self-oscillators and supports our theoretical findings [80]. This section outlines the theoretical framework for an electrically driven LCE self-oscillator, starting with the dynamic model that describes its behavior, and leads to the derivation of the general governing equations. Following this, we introduce the model of an electrothermally responsive LCE, apply nondimensionalization to simplify the equations, and present the solution method.

### 2.1. Dynamic Model of Electrically Driven LCE Self-Oscillator

**Figure 1:**
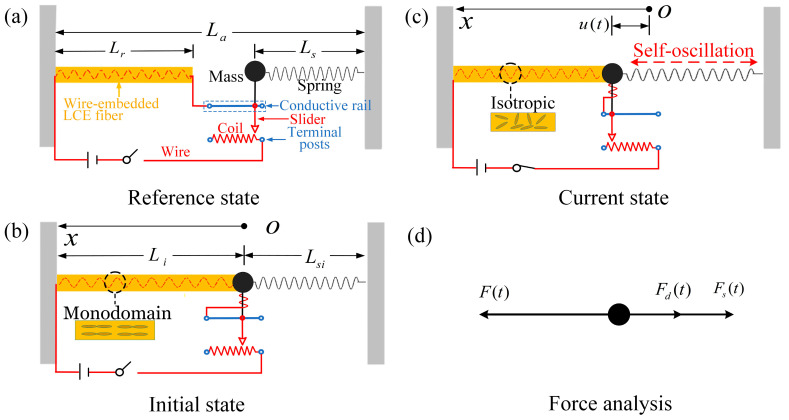
Schematic of electrically driven LCE self-oscillator based on rheostat feedback mechanism. (**a**) Reference state. (**b**) Initial state. (**c**) Current state. (**d**) Force analysis. Through feedback from the motion-dependent rheostat, the mass maintains periodic horizontal oscillation.

Figure 1 is a schematic illustration of an electrically driven LCE self-oscillator based on the rheostat feedback mechanism which consists of an LCE fiber embedded with resistive wires, a mass, a spring, a nonlinear rheostat, and a constant voltage source. In the reference state (Figure 1a), the connection between the LCE and the rheostat can be observed. The lead wire is connected from the positive terminal of the power supply to the left end of the LCE and runs through to the right end of the LCE. It is then connected to the left terminal post of the conductive rail of the rheostat. The lead wire then enters the right terminal post of the coil and returns to the switch, which is connected to the negative terminal of the power supply, thus completing the circuit. The switch is open, and the circuit is not connected. The LCE fiber of original length $L_{r}$ is anchored at the left boundary. A mass rigidly connected to the slider is attached to the left end of a spring of length $L_{s}$ fixed at the right boundary. The distance between the two anchored ends is $L_{a}$.

The initial state of the system is depicted in Figure 1b. At this moment, the switch is still open, and the circuit is not connected. In LCE fibers, the rod-like molecules are oriented along the fiber’s axis, a structural arrangement referred to as the monodomain configuration. The right terminal of the LCE is connected to the left side of the spring, and the lengths of the spring and LCE are $L_{i}$ and $L_{si}$, respectively. The mass’s initial position is defined as the origin o, with the coordinate framework established in the positive orientation to the left. Figure 1c illustrates the self-oscillating system under a constant voltage source, with the instantaneous displacement u(t) of the mass shown in its current state. When the switch is closed, the LCE begins to absorb the electrothermal energy generated by the embedded wires. The rod-like molecules in the LCE shift from a monodomain state to an isotropic state, causing the LCE to contract. This simultaneously causes the slider and the end of the LCE to move in synchronization. By changing the resistance of the circuit, the electrothermal energy generated by the current in the LCE is altered, inducing self-oscillation [81]. For simplicity, the gravity on the mass can be ignored. As shown in the force analysis of Figure 1d, the mass moves under the influence of the internal tension F(t) from the LCE, the elastic force $F_{s}(t)$ from the spring, and the damping force $F_{d}(t)$; the mechanical control equation for the mass can be derived by using Newton’s second law:

(1) $$m\ddot{u}(t)=F(t)-F_{s}(t)-F_{d}(t),$$

where m is the mass and $\ddot{u}(t)$ is the acceleration of the mass.

Assuming that the force exerted by the spring attached to the mass varies linearly with its deformation, the spring force is

(2) $$F_{s}(t)=j\left[L_{a}-L_{i}+u(t)-L_{s}\right],$$

where j is the elastic coefficient of the spring.

The viscoelastic properties of the LCE are considered small in this study, and its impact on the system is negligible and therefore disregarded in the analysis [82]. Assuming that the damping force consists of two components, i.e., a linear damping term proportional to velocity and a nonlinear additional damping term dependent on the absolute value of velocity, in Equation (1), the damping force is

(3) $$F_{d}(t)=(C_{1}+C_{2}\left|\dot{u}(t)\right|)\dot{u}(t),$$

where $C_{1}$ is the first damping coefficient and $C_{2}$ is the second damping coefficient.

In this model, since the deformations of the LCE involved are small, it could be postulated that the internal tension from the LCE changes linearly with the stretching; then, the internal tension within the LCE can be calculated from the elongation of the LCE and the spring:

(4) $$F\left(t\right)=k\left[\frac{j(L_{a}-L_{i}-L_{s})}{k}-u\left(t\right)-L_{i}\varepsilon \left(t\right)\right],$$

where k denotes the elasticity coefficient of the LCE and ε(t) signifies the electrically driven strain of the LCE. Here, the elasticity coefficient is assumed to be independent of the temperature.

Typically, the connection between electrically driven deformation and temperature is intricate. We posit that $\varepsilon \left(t\right)$ is directly proportional to the temperature difference $T\left(t\right)$. As a result, we can express this relationship as follows:

(5) $$\varepsilon \left(t\right)=-\alpha T\left(t\right),$$

where α is the contraction coefficient. α>0 indicates the negative strain and electrically driven contraction, while α<0 indicates electrically driven expansion.

### 2.2. Electrothermally Responsive LCE Model

We further assume that the radius R of the LCE fiber is much smaller than the length $L_{i}$. In the case of extremely thin fibers, it is reasonable to presume that the temperature distribution across the fiber’s thickness is uniform. In the experiments cited by references [17,83,84], typical parameters are as follows: heat transfer coefficient $k_{c}=10{\;W/m}^{2}/K$, thermal conductivity λ=0.1 W/m/K, and $R=10^{-5}\;m$. With these values, the Biot number, defined by $B_{i}\;=k_{c}\;R/\lambda$, is calculated to be $B_{i}\;=10^{-3}$. The small Biot number indicates that heat transfer in the thickness direction is very fast due to the short diffusion distance. In the electrically driven LCE self-oscillator, the quantity of energy converted from electrical to thermal and subsequently distributed throughout the LCE is captured by the electrothermal intensity Q. The electrothermal intensity Q refers to the energy transferred per unit time ΔT, which depends on the temperature contrast between the electrothermally responsive LCE and its surroundings. The temperature of LCE fiber can be determined by the following formula:

(6) $$\frac{d\left(T_{a}-T_{ext}\right)}{dt}=\frac{Q-k_{c}\left(T_{a}-T_{ext}\right)}{\rho_{c}},$$

where $\rho_{c}$ denotes the specific heat capacity, $k_{c}$ represents the heat transfer coefficient, $T_{a}$ is the absolute temperature of the LCE fiber, and $T_{\mathrm{ext}}$ is the surrounding temperature.

By introducing the relative temperature T, i.e., $T=T_{a}-T_{\mathrm{ext}}$, we can rewrite Equation (6) as follows:

(7) $$\frac{dT}{dt}=\frac{Q-k_{c}T}{\rho_{c}}.$$

To calculate the evolution of temperature with time, Equation (7) can be further expressed as

(8) $$\frac{dT}{dt}=\frac{T_{L}-T}{\tau },$$

where $T_{L}=\frac{Q}{k_{c}}$ represents the maximum temperature difference that the electrothermally responsive LCE can achieve under steady electrothermal intensity and $\tau =\frac{\rho }{k_{c}}$ represents thermal relaxation time, which reflects the rate of heat exchange between the electrothermally responsive LCE and its surroundings. A higher τ signifies that the LCE needs more time to attain the maximum temperature difference $T_{L}$. We aim to select a smaller value of τ to meet the rapid response characteristics of this electrothermally responsive LCE model.

Due to rapid heat exchange, the temperature inside the LCE remains uniform. Assuming that the conductive coil inside the LCE is regarded as a purely resistive element, the feedback adjustment of the rheostat ensures that the current always varies linearly with the displacement of the mass. Consequently, we can establish that the electrothermal intensity maintains the following linear relationship:

(9) $$Q(t)=-\beta u(t)+Q_{0},$$

where β is the electrothermal intensity coefficient and $Q_{0}$ is the initial electrothermal intensity. It can be deduced that as the LCE contracts more, it absorbs less electrothermal energy, and as it relaxes and begins to expand, the absorption of electrothermal energy increases, leading to the periodic oscillation of the mass.

### 2.3. Nondimensionalization

To convert the above control equations into dimensionless form, we introduce the following set of dimensionless parameters:

$$\bar{\tau }=\tau /\sqrt{m/k},\;\bar{t}=t/\sqrt{m/k},\;\bar{j}=j/k,\;\bar{\alpha }=\alpha T_{ext},\;{\bar{C}}_{1}=C_{1}/\sqrt{km},\;{\bar{C}}_{2}=C_{2}L_{i}/m,\;{\bar{L}}_{a}=L_{a}/L_{i},\;{\bar{L}}_{s}=L_{s}/L_{i},\;\bar{u}\left(t\right)=u\left(t\right)/L_{i},\;\bar{F}\left(t\right)=F\left(t\right)/kL_{i},\;{\bar{F}}_{s}\left(t\right)=F_{s}\left(t\right)/kL_{i},\;\bar{T}=T/T_{ext},\;\bar{Q}(t)=Q/k_{c}T_{ext},\;{\bar{Q}}_{0}=Q_{0}/k_{c}T_{ext},\;\bar{\beta }=\beta L_{i}/k_{c}T_{ext}.$$

We then obtain the nondimensional form of Equation (1) as follows:

(10) $$\bar{\ddot{u}}(\bar{t})=\bar{F}(\bar{t})-\bar{F_{s}}(\bar{t})-\bar{F_{d}}(\bar{t}),$$

The spring force nondimensionalization of Equation (2) is

(11) $$\bar{F_{s}}\left(\bar{t}\right)=\bar{j}\left[\bar{L_{a}}-1+\bar{u}\left(\bar{t}\right)-\bar{L_{s}}\right].$$

Similarly, Equation (3) can be nondimensionalized as

(12) $${\bar{F}}_{d}(\bar{t})=({\bar{C}}_{1}+{\bar{C}}_{2}\left|\bar{\dot{u}}(\bar{t})\right|)\bar{\dot{u}}(\bar{t}).$$

The dimensionless form of Equation (4) for LCE tension is

(13) $$\bar{F}\left(\bar{t}\right)=\left[\bar{j}(\bar{L_{a}}-1-\bar{L_{s}})-\bar{u}\left(\bar{t}\right)-\varepsilon \left(t\right)\right],$$

where $\varepsilon \left(t\right)$ is expressed as

(14) $$\varepsilon \left(t\right)=-\bar{\alpha }\bar{T}(t).$$

The expression for Equation (7) in dimensionless form is

(15) $$\frac{d\bar{T}}{d\bar{t}}=\frac{\bar{Q}-\overset{\_\_}{T}}{\bar{\tau }},$$

where $\bar{Q}$ is further articulated as

(16) $$\bar{Q}(\bar{t})=-\bar{\beta }\bar{u}(\bar{t})+{\bar{Q}}_{0}.$$

By combining Equations (10)–(14), we can write

(17) $$\bar{\ddot{u}}\left(\bar{t}\right)=(-1-\bar{j})\bar{u}\left(\bar{t}\right)+\bar{\alpha }\bar{T}\left(\bar{t}\right)-{\bar{C}}_{1}\bar{\dot{u}}\left(\bar{t}\right)-{\bar{C}}_{2}\left|\bar{\dot{u}}\left(\bar{t}\right)\right|\bar{\dot{u}}\left(\bar{t}\right).$$

To solve the equations mentioned above in Equations (13)–(17), we can employ the Runge–Kutta method implemented in MATLAB 2023a by specifying the parameters $\bar{\alpha }$, $\bar{\beta }$, ${\bar{Q}}_{0}$, ${\bar{C}}_{1}$, ${\bar{C}}_{2}$, $\bar{\tau }$, $\bar{j}$, ${\bar{L}}_{a}$, and ${\bar{L}}_{s}$. Before commencing the computation, we pre-select a time step Δt of 0.0001. According to Equations (15) and (16), the current temperature ${\bar{T}}_{n}$ can be used to calculate the current strain $\varepsilon_{n}$ from Equation (14) and the current position ${\bar{u}}_{n}$ from Equation (17). Subsequently, $\varepsilon_{n}$ and ${\bar{u}}_{n}$ are substituted into Equation (13) to deduce the LCE tension ${\bar{F}}_{n}$. As the next iteration begins, we again calculate the next temperature ${\bar{T}}_{n+1}$ from Equations (15) and (16); this enables us to calculate the next moment’s strain $\varepsilon_{n+1}$ from Equation (14) and the next moment’s position ${\bar{u}}_{n+1}$ from Equation (17), while simultaneously deriving the LCE tension ${\bar{F}}_{n+1}$. Thus, through the iterative calculations of the variables above, the vibrational properties of the electrically driven LCE self-oscillator can be observed.

## 3. Asymptotic Analysis

Throughout this section, we apply the earlier-discussed general governing equation to formulate the asymptotic equation pertaining to the electrically driven LCE self-oscillator, employing an asymptotic substitution with a small parameter $\bar{\tau }$ related to an increase in the heat transfer coefficient or a decrease in the heat capacity. When $\bar{\tau }<<1$, the following relationship can be established:

(18) $$\bar{T}={\bar{T}}^{\left(0\right)}+\bar{\tau }{\bar{T}}^{\left(1\right)}+o\left({\bar{\tau }}^{2}\right).$$

By substituting Equation (18) into Equation (15) and neglecting the infinitesimal term $o\left({\bar{\tau }}^{2}\right)$, we obtain

(19) $$\bar{\tau }\frac{d{\bar{T}}^{\left(0\right)}}{d\bar{t}}+{\bar{\tau }}^{2}\frac{d{\bar{T}}^{\left(1\right)}}{d\bar{t}}=\bar{Q}-{\bar{T}}^{\left(0\right)}-\bar{\tau }{\bar{T}}^{\left(1\right)}.$$

By comparing the coefficients of $\bar{\tau }$ from Equation (19), we deduce that

(20) $${\bar{T}}^{\left(0\right)}=\bar{Q}.$$

Consequently, we derive

(21) $${\bar{T}}^{\left(1\right)}=-\frac{d\bar{Q}}{d\bar{t}}.$$

Substituting Equations (20) and (21) into Equation (18) yields

(22) $$\bar{T}=\bar{Q}-\bar{\tau }\frac{d\bar{Q}}{d\bar{t}}.$$

Substituting the linear electrothermal intensity expression from Equation (16) into Equation (22) gives

(23) $$\bar{T}={\bar{Q}}_{0}-\bar{\beta }\bar{u}(\bar{t})+\bar{\beta }\bar{\tau }\frac{d\bar{u}(\bar{t})}{d\bar{t}}.$$

Putting Equation (23) into Equation (17) results in the asymptotic equation

(24) $$\bar{\ddot{u}}(\bar{t})=(-1-\bar{j})\bar{u}(\bar{t})+\bar{\alpha }{\bar{Q}}_{0}-\bar{\alpha }\bar{\beta }\bar{u}(\bar{t})+\bar{\alpha }\bar{\beta }\bar{\tau }\bar{\dot{u}}(\bar{t})-({\bar{C}}_{1}+{\bar{C}}_{2}\left|\bar{\dot{u}}(\bar{t})\right|)\bar{\dot{u}}(\bar{t}).$$

Differing from governing Equations (13)–(17), Equation (24) represents the asymptotic equation for the electrically driven LCE self-oscillator. By specifying the parameters $\bar{\alpha }$, $\bar{\beta }$, ${\bar{Q}}_{0}$, ${\bar{C}}_{1}$, ${\bar{C}}_{2}$, $\bar{\tau }$, and $\bar{j}$, we can further apply the Runge–Kutta method to obtain the self-oscillation’s time history curve according to Equation (24). Moreover, the asymptotic equation provides an efficient, convenient, and precise approach while avoiding the complexity of needing to solve multiple dynamic equations.

## 4. Two Motion Phases and Self-Oscillation Mechanism

By employing the general governing equation alongside the asymptotic equation discussed earlier, this section initiates a numerical investigation into the behaviors of the electrically driven LCE self-oscillator via the rheostat feedback mechanism. We identify two distinct motion phases, i.e., the static phase and the self-oscillating phase, and further delve into the mechanisms underlying self-oscillation.

### 4.1. Two Motion Phases

**Figure 2:**
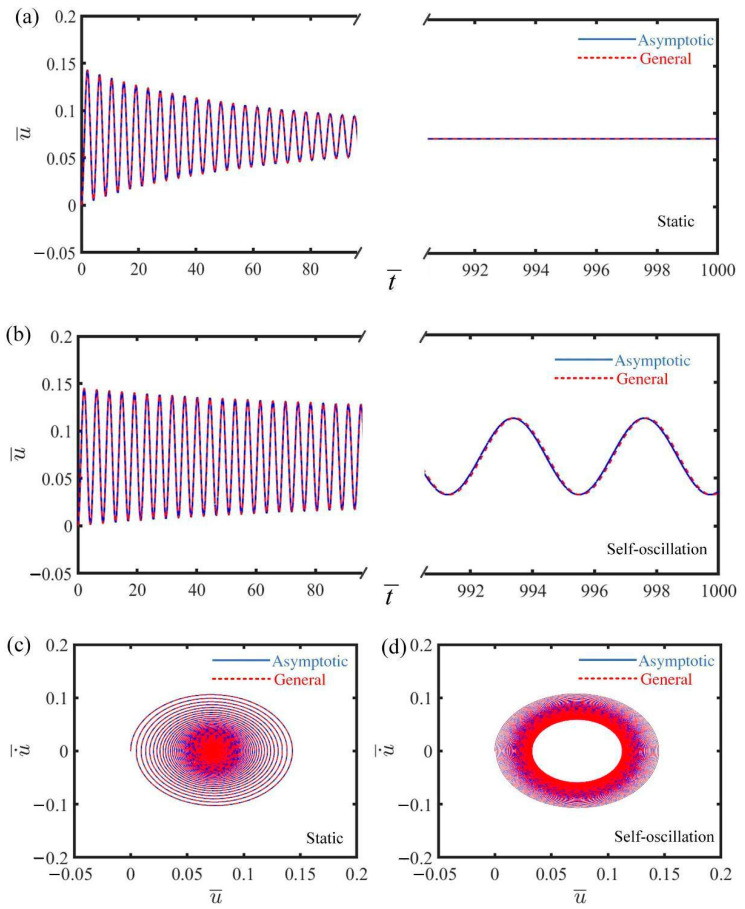
Two motion phases of electrically driven LCE self-oscillator. (**a**,**c**) Displacement–time history curve and phase trajectory plot in static phase, where ${\bar{C}}_{1}=0.025$. (**b**,**d**) Displacement–time history curve and phase trajectory plot in self-oscillating phase, where ${\bar{C}}_{1}=0$. All other parameters remain consistent: $\bar{\alpha }=0.2$, $\bar{\tau }=0.01$, $\bar{\beta }=5$, $\bar{j}=0.2$, ${\bar{C}}_{2}=0.2$, ${\bar{L}}_{a}=1.9$, ${\bar{L}}_{s}=0.6$, and ${\bar{Q}}_{0}=0.8$. By varying the first damping coefficients, two distinct motion phases are capable of being realized: the static phase and the self-oscillating phase.

Figure 2 illustrates the displacement–time history curve and phase trajectory plot for the mass under two distinct first damping coefficients, depicting both the static and self-oscillating phases of the electrically driven LCE self-oscillator. In Figure 2a,c, the displacement–time history curve and phase trajectory plot of the mass are presented for the case where ${\bar{C}}_{1}=0.025$. The displacement initially exhibits noticeable fluctuations, gradually approaching a specific position as time progresses, as shown in Figure 2a. The velocity of the mass in Figure 2c gradually decreases, eventually reaching zero at the corresponding position in Figure 2a, further confirming the static phase. Figure 2b,d reveal the displacement–time history curve and phase trajectory plot of the mass for the case where ${\bar{C}}_{1}=0$. From Figure 2b, we can see that the displacement exhibits periodic characteristics as time progresses. Additionally, the steady limit cycle formed in the velocity trajectory plot of Figure 2d also indicates the self-oscillating phase. Overall, the results from Figure 2 above show that the numerical calculations for the governing and asymptotic equations exhibit a high degree of similarity, validating the accuracy of the results.

### 4.2. Self-Oscillation Mechanism

**Figure 3:**
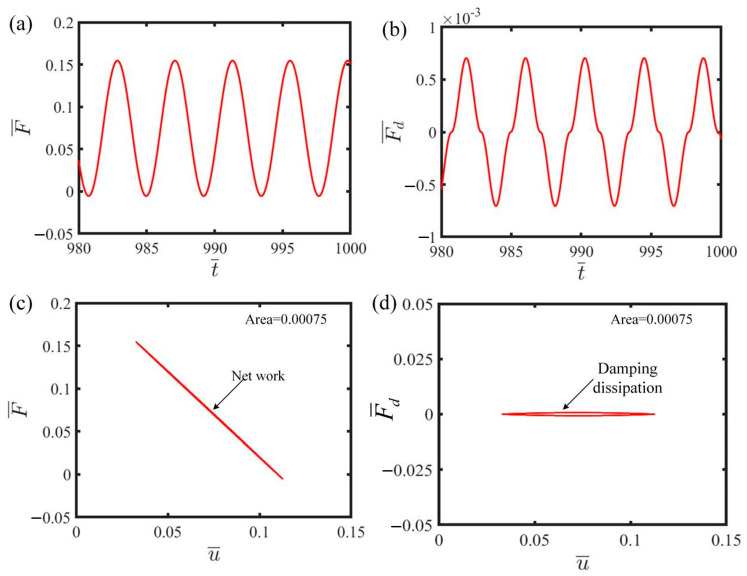
The energy compensation mechanism of the electrically driven LCE self-oscillator. (**a**) LCE tension as a function of time. (**b**) Damping force as a function of time. (**c**) LCE tension as a function of displacement. (**d**) Damping force as a function of displacement. The work performed by the LCE tension compensates for the energy dissipated by damping, thereby sustaining self-oscillation.

Figure 3 shows the energy compensation mechanism of the electrically driven LCE self-oscillator based on the self-oscillating phase depicted in Figure 2b,d. Figure 3a describes the cyclical fluctuation in tension in the LCE throughout the duration, showcasing the self-oscillation of the LCE self-oscillator. Figure 3b shows that the damping force also varies periodically with time, exhibiting a cyclical trend. Figure 3c illustrates how the LCE tension changes with the displacement of the mass. Within a specific period, the formation of a closed loop occurs, and the area enclosed by this curve, measuring 0.00075, accurately signifies the net work performed by the LCE tension. Similarly, in Figure 3d, during the same specific period mentioned above, the curve of damping force versus displacement also forms a closed loop, and the enclosed area of 0.00075 represents the energy dissipated by damping. This equilibrium between the energy generated by the LCE tension and the energy dissipated by damping ensures that the LCE self-oscillator remains in a steady self-oscillating phase.

## 5. Multi-Scale Analysis of Asymptotic Equations

Following the discovery of the two motion phases discussed earlier, the circumstances under which self-oscillations occur in the system, as well as the calculations of their amplitudes and frequencies, are noteworthy. This section utilizes the Hurwitz criterion to obtain an analytical formula for the bifurcation points and, through multi-scale analysis, derives analytical formulas for the amplitude and frequency in the self-oscillation of the LCE self-oscillator.

### 5.1. Hurwitz Criterion

To analyze the conditions for self-oscillations, we aim to identify the bifurcation points of the system, thereby utilizing the Hurwitz criterion as part of our solution process. The process starts with a review of Equation (24), where we simplify it by combining the terms according to their displacement orders and consolidating the coefficients:

(25) $$\bar{\ddot{u}}(\bar{t})+(-\bar{\alpha }\bar{\beta }\bar{\tau }+{\bar{C}}_{1})\left[\bar{\dot{u}}(\bar{t})+\frac{{\bar{C}}_{2}}{-\bar{\alpha }\bar{\beta }\bar{\tau }+{\bar{C}}_{1}}\left|\bar{\dot{u}}(\bar{t})\right|\bar{\dot{u}}(\bar{t})\right]+(\bar{j}+1+\bar{\alpha }\bar{\beta })\bar{u}(\bar{t})-\bar{\alpha }{\bar{Q}}_{0}=0.$$

By defining the parameters $a_{1}=-{\bar{C}}_{2}/\left(-\bar{\alpha }\bar{\beta }\bar{\tau }+{\bar{C}}_{1}\right)$, $a_{2}=\bar{j}+1+\bar{\alpha }\bar{\beta }$, $a_{3}=-\bar{\alpha }{\bar{Q}}_{0}$, and $\varepsilon_{0}=\bar{\alpha }\bar{\beta }\bar{\tau }-{\bar{C}}_{1}$, Equation (25) can be written as

(26) $$\bar{\ddot{u}}(\bar{t})-\varepsilon_{0}\left[\bar{\dot{u}}(\bar{t})-a_{1}\left|\bar{\dot{u}}(\bar{t})\right|\bar{\dot{u}}(\bar{t})\right]+a_{2}\bar{u}(\bar{t})+a_{3}=0.$$

Substituting $x\left(\bar{t}\right)=\bar{u}\left(\bar{t}\right)+a_{3}/a_{2}$ into Equation (26) yields

(27) $$\ddot{x}-\varepsilon_{0}\left[\dot{x}-a_{1}\left|\dot{x}\right|\dot{x}\right]+a_{2}x=0.$$

By using the method of linear perturbation, the linearized Equation (27) is expressed as

(28) $$\ddot{x}-\varepsilon_{0}\dot{x}+a_{2}x=0.$$

The determinant of the Hurwitz characteristic equation for Equation (28) at each order is represented by $H_{1}=\left|1\right|$, $H_{2}=\left|-\varepsilon_{0}\right|$, and $H_{3}=\left|\begin{array}{cc} -\varepsilon_{0} & 0\\ 1 & a_{2} \end{array}\right|$. Based on the Hurwitz criterion [85], it is required that $H_{1}>0$, $H_{2}>0$ and $H_{3}>0$. To ensure asymptotic stability in the system, it is necessary that $a_{2}>0$ and $\varepsilon_{0}<0$. Hence, the Hurwitz criterion $\varepsilon_{0}$ can be formulated as

(29) $$\varepsilon_{0}=\bar{\alpha }\bar{\beta }\bar{\tau }-{\bar{C}}_{1}.$$

**Figure 4:**
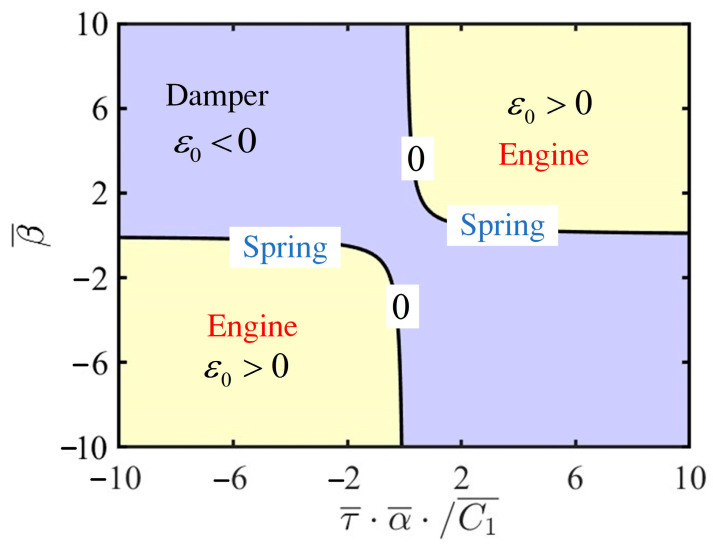
The contour plot for $\varepsilon_{0}$ based on the parameters $\bar{\beta }$ and $\bar{\tau }\bar{\alpha }/{\bar{C}}_{1}$, showing the three distinct behaviors of the LCE self-oscillator. These behaviors include damping, engine, and spring characteristics.

From Equation (29), it can be inferred that several parameters of the LCE self-oscillator, including contraction coefficient $\bar{\alpha }$, thermal relaxation time $\bar{\tau }$, thermal intensity coefficient $\bar{\beta }$, and the first damping coefficient ${\bar{C}}_{1}$, collectively influence the value of $\varepsilon_{0}$. The analytical expression for the bifurcation points reveals three distinct behaviors of the LCE self-oscillator: damping, engine, and spring, as illustrated in Figure 4. For $\varepsilon_{0}<0$, the LCE self-oscillator remains static, representing the damping mechanism responsible for energy dissipation. When $\varepsilon_{0}>0$, the LCE self-oscillator enters self-oscillation, acting like an engine by absorbing energy from the constant voltage source. Furthermore, the bifurcation points are present in the system at $\varepsilon_{0}=0$, and the LCE self-oscillator exhibits an elastic spring.

### 5.2. Amplitude and Frequency

The previously discussed approaches of using Runge–Kutta iterations to study the dynamic behavior of the LCE self-oscillator are noted. Furthermore, this section primarily employs multi-scale mathematical methods [86,87] to derive formulas, providing direct and explicit expressions for both amplitude and frequency. We can rewrite Equation (27) as

(30) $$\ddot{x}+\omega_{0}^{2}x=\varepsilon f\left(x,\dot{x}\right),$$

where $f(x,\dot{x})=\dot{x}-a_{1}|\dot{x}|\dot{x}$ and $\omega_{0}=\sqrt{a_{2}}$; furthermore, we express the solution of this equation in the following form:

(31) $$x(t)=x_{0}(I_{0},I_{1},I_{2})+\varepsilon_{0}x_{1}(I_{0},I_{1},I_{2})+{\varepsilon_{0}}^{2}x_{2}(I_{0},I_{1},I_{2}),$$

where $T_{n}=\varepsilon_{0}^{n}t(n=0,1,2)$. In terms of $T_{n}$, the time derivatives become

(32) $$\dot{x}(t)=D_{0}+\varepsilon_{0}D_{1}+{\varepsilon_{0}}^{2}D_{2},$$

(33) $$\ddot{x}(t)={D_{0}}^{2}+2\varepsilon_{0}D_{0}D_{1}+{\varepsilon_{0}}^{2}({D_{1}}^{2}+2D_{0}D_{2}),$$

where $D_{n}=\frac{\partial }{\partial T_{n}}(n=0,1,2)$. By substituting Equations (31)–(33) into Equation (30) and comparing the coefficients of the same powers of $\varepsilon_{0}$, we obtain

(34) $${D_{0}}^{2}x_{0}+{\omega_{0}}^{2}x_{0}=0,$$

(35) $${D_{0}}^{2}x_{1}+{\omega_{0}}^{2}x_{1}=-2D_{0}D_{1}x_{0}+f(x_{0},D_{0}x_{0}),$$

By continuing to substitute Equations (34) and (35) back into Equation (30), we obtain the first-order approximate solution to Equation (30) as

(36) $$x=a\cos(\omega_{0}t+\theta_{0})+o\left(\varepsilon_{0}\right),$$

where $a=\frac{3\pi }{8\omega_{0}a_{1}}+\frac{1}{e^{\frac{1}{2}(\varepsilon_{0}t+\omega_{0})}}$ and $\theta =\theta_{0}$.

By substituting $x=\bar{u}+a_{3}/a_{2}$ into Equation (36), the resolution of the governing equation is

(37) $$\bar{u}=(\frac{3\pi }{8\sqrt{a_{2}}a_{1}}+\frac{1}{e^{\frac{1}{2}(\varepsilon_{0}t+\omega_{0})}})(\cos(\sqrt{a_{2}}t+\theta_{0})-\frac{a_{3}}{a_{2}}+ο(\varepsilon_{0}),$$

where $a_{1}=\frac{-{\bar{C}}_{2}}{\left(-\bar{\alpha }\bar{\beta }\bar{\tau }+{\bar{C}}_{1}\right)}$, $a_{2}=\bar{j}+1+\bar{\alpha }\bar{\beta }$, and $a_{3}=-\bar{\alpha }{\bar{Q}}_{0}$.

By substituting $a_{1}$, $a_{2}$, and $a_{3}$ into Equation (37), the amplitude and frequency can be written as follows:

(38) $$A=\frac{3\pi }{8\sqrt{\bar{j}+1+\bar{\alpha }\bar{\beta }}\cdot \frac{-{\bar{C}}_{2}}{\left(-\bar{\alpha }\bar{\beta }\bar{\tau }+{\bar{C}}_{1}\right)}},$$

(39) $$f=\sqrt{\bar{j}+1+\bar{\alpha }\bar{\beta }}.$$

Equations (38) and (39) are analytical expressions for the amplitude and frequency associated with the self-oscillation of the LCE self-oscillator. The amplitude and frequency are determined by using the parameters $\bar{\alpha }$, $\bar{\beta }$, $\bar{\tau }$, $\bar{j}$, ${\bar{C}}_{1}$, and ${\bar{C}}_{2}$. Using these analytical solutions facilitates the immediate assessment of the variations in amplitude and frequency in response to varying parameters, thus streamlining the analysis of self-oscillation. Subsequently, based on the results obtained, the following sections will focus on how different parameters specifically influence the behavior of the LCE self-oscillator.

## 6. Parameter Analysis

Based on Equation (29) derived from the Hurwitz criterion $\varepsilon_{0}$, we can categorize the parameters into two main groups. The first group includes the parameters $\bar{\alpha }$, ${\bar{C}}_{1}$, $\bar{\tau }$, and $\bar{\beta }$, which influence the value of $\varepsilon_{0}$. The second group consists of the parameters $\bar{j}$, ${\bar{Q}}_{0}$, ${\bar{C}}_{2}$, ${\bar{L}}_{a}$, and ${\bar{L}}_{s}$, which do not appear in the equation. This section discusses how two groups of parameters impact self-oscillation amplitude and frequency by using numerical solutions from both the governing and asymptotic equations, as well as multi-scale analytical solutions. The results obtained from these approaches demonstrate a high degree of consistency.

### 6.1. Effects of Parameters Relevant to Bifurcation Points

**Figure 5:**
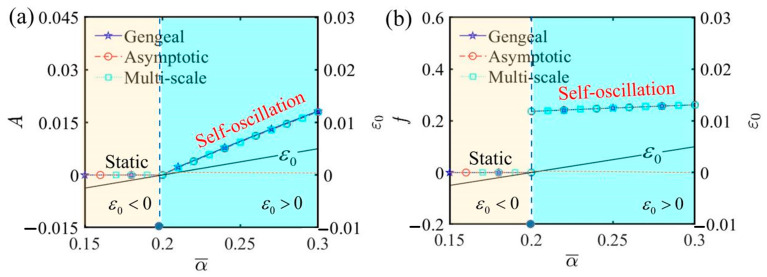
The influence of the contraction coefficient $\bar{\alpha }$ on self-oscillation. (**a**) Amplitude and $\varepsilon_{0}$. (**b**) Frequency and $\varepsilon_{0}$. In the self-oscillating phase, both amplitude and frequency generally show nearly linear increases with the increase in $\bar{\alpha }$.

Figure 5 shows the impact of the contraction coefficient on the self-oscillation behavior of the LCE self-oscillator. The remaining parameters in the system are defined as ${\bar{C}}_{1}=0.01$, $\bar{\tau }=0.01$, $\bar{\beta }=5$, $\bar{j}=0.2$, ${\bar{Q}}_{0}=0.8$, ${\bar{C}}_{2}=0.2$, ${\bar{L}}_{a}=1.9$, and ${\bar{L}}_{s}=0.8$. From these plots, it is evident that with certain parameter settings, self-oscillation is initiated at a critical threshold condition $\bar{\alpha }=0.2$. When the value of the contraction coefficient falls below this critical threshold and $\varepsilon_{0}<0$, the LCE self-oscillator transitions into a static phase. Conversely, when the value of the contraction coefficient exceeds the critical threshold and $\varepsilon_{0}>0$, the LCE self-oscillator transitions into a self-oscillating phase. Figure 5a,b plot the amplitude and frequency across various contraction coefficients, respectively. As the value of $\bar{\alpha }$ rises, both the metrics also increase. This phenomenon occurs because, when other variables are held constant, a rise in the contraction coefficient leads to greater contraction strain produced by the LCE fiber. Consequently, this leads to increased deformation and net work generated within the LCE fiber, resulting in more intense self-oscillation effects. As a result, raising the contraction coefficient of the LCE fiber could greatly boost the effectiveness in applications like actuators and energy harvesting.

**Figure 6:**
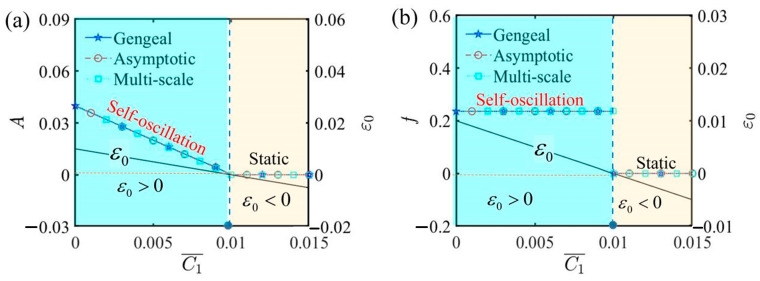
The influence of the first damping coefficient ${\bar{C}}_{1}$ on self-oscillation. (**a**) Amplitude and $\varepsilon_{0}$. (**b**) Frequency and $\varepsilon_{0}$. As the first damping coefficient increases, the amplitude tends to decrease, while the frequency remains constant. The value of $\varepsilon_{0}$ gradually decreases until it converges at the critical point ${\bar{C}}_{1}=0.01$, at which all three become zero.

Figure 6 depicts how the first damping coefficient influences the self-oscillation of the LCE self-oscillator. For the computation, $\bar{\alpha }=0.2$, $\bar{\tau }=0.01$, $\bar{\beta }=5$, $\bar{j}=0.2$, ${\bar{Q}}_{0}=0.8$, ${\bar{C}}_{2}=0.2$, ${\bar{L}}_{a}=1.9$, and ${\bar{L}}_{s}=0.8$ are assigned. With certain parameter settings, self-oscillation is initiated at a critical threshold condition ${\bar{C}}_{1}=0.01$. When the first damping coefficient is beneath this pivotal limit and $\varepsilon_{0}<0$, the self-oscillator transitions into a static phase. Once the first damping coefficient surpasses the critical threshold and $\varepsilon_{0}>0$, the LCE self-oscillator transitions into a self-oscillating phase. From Figure 6a, an increased damping coefficient dampens the self-oscillation of the LCE self-oscillator, gradually reducing the amplitude until it approaches zero. From Figure 6b, the first damping coefficient fails to significantly influence the frequency, except in a specific case where ${\bar{C}}_{1}=0.01$. This phenomenon suggests that a larger first damping coefficient results in the LCE self-oscillator absorbing less energy, resulting in a reduction in amplitude; however, the self-oscillation period remains unchanged. Therefore, selecting a smaller first damping coefficient may greatly enhance the capability of the LCE self-oscillator, thereby enhancing its efficiency and functionality in practical applications.

**Figure 7:**
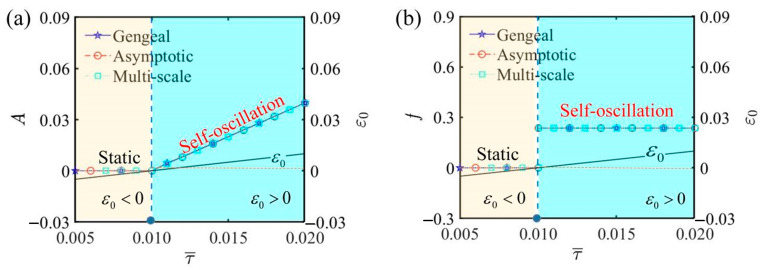
The influence of the thermal relaxation time $\bar{\tau }$ on self-oscillation. (**a**) Amplitude and $\varepsilon_{0}$. (**b**) Frequency and $\varepsilon_{0}$. With the increase in thermal relaxation time, the LCE self-oscillator transitions from a static phase to a self-oscillating phase, with the amplitude gradually increasing and the frequency remaining largely unaffected.

Figure 7 illustrates the influence of thermal relaxation time on the self-oscillation of the LCE self-oscillator under the given parameters $\bar{\alpha }=0.2$, ${\bar{C}}_{1}=0.01$, $\bar{\beta }=5$, $\bar{j}=0.2$, ${\bar{Q}}_{0}=0.8$, ${\bar{C}}_{2}=0.2$, ${\bar{L}}_{a}=1.9$, and ${\bar{L}}_{s}=0.8$. When the thermal relaxation time is $\bar{\tau }<0.01$, the LCE self-oscillator exhibits a static phase. When $\bar{\tau }>0.01$, the LCE self-oscillator transitions into a self-oscillating phase. Figure 7a shows that the amplitude of self-oscillation augments with the thermal relaxation time. It happens because a longer thermal relaxation time allows the LCE fiber to take in more electrothermal energy, subsequently transforming it into greater kinetic energy, resulting in an amplitude increase. As illustrated in Figure 7b, the impact of the thermal relaxation time on the frequency of self-oscillation is negligible. The results indicate that creating thinner LCE fibers can extend the thermal relaxation time, thereby enhancing the efficacy. By increasing the parameter $\bar{\tau }$, the LCE fiber’s responsiveness to temperature changes improves, making it more effective in various applications.

**Figure 8:**
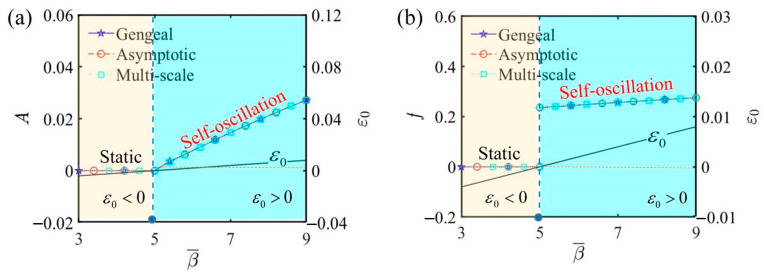
The influence of the electrothermal intensity coefficient $\bar{\beta }$ on self-oscillation. (**a**) Amplitude and $\varepsilon_{0}$. (**b**) Frequency and $\varepsilon_{0}$. The critical value $\bar{\beta }=5$ determines the motion phases of the LCE self-oscillator: when $\bar{\beta }<5$, it remains static, whereas when $\bar{\beta }<5$, it enters self-oscillation. Additionally, the electrothermal intensity coefficient increases, leading to a rise in both amplitude and frequency.

Figure 8 illustrates the amplitude and frequency of self-oscillation under different electrothermal intensity coefficients, highlighting how the electrothermal intensity coefficient affects self-oscillation. In this process, we set $\bar{\alpha }=0.2$, ${\bar{C}}_{1}=0.01$, $\bar{\tau }=0.01$, $\bar{j}=0.2$, ${\bar{Q}}_{0}=0.8$, ${\bar{C}}_{2}=0.2$, ${\bar{L}}_{a}=1.9$, and ${\bar{L}}_{s}=0.8$. Figure 8a illustrates that an increase in the electrothermal intensity coefficient leads to a rise in amplitude. Figure 8b illustrates that a higher electrothermal intensity coefficient correlates with an increase in the frequency. From $\bar{\beta }=\beta L_{i}/k_{c}T_{ext}$, it can be seen that the effect of the length of the LCE fiber $L_{i}$ on the frequency is the same as that of the electrothermal intensity coefficient. As $L_{i}$ increases, the frequency also increases. This is primarily due to the enhanced electrothermal energy absorption and the system’s mechanical response to this energy. The longer fiber absorbs more energy, leading to more frequent oscillations to maintain the balance between energy input and dissipation. An augmented $\bar{\beta }$ results in greater absorption of electrothermal energy by the LCE fiber, thereby increasing the amplitude of self-oscillation. Conversely, when $\bar{\beta }$ is lower, the LCE fiber absorbs less electrothermal energy, resulting in a smaller self-oscillation amplitude and placing the system in a reduced-energy state. In this state, the vibration may either destabilize, weaken, or fail to sustain self-oscillation. Thus, altering the electrothermal intensity coefficient facilitates the precise adjustment of the oscillatory behavior of the LCE self-oscillator to align with the demands of diverse implementations.

### 6.2. Effects of Parameters Irrelevant to Bifurcation Points

**Figure 9:**
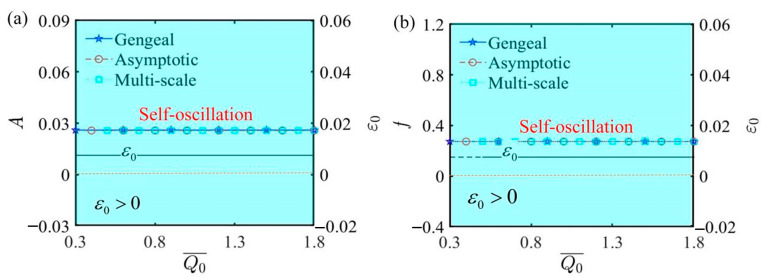
The influence of the initial electrothermal intensity ${\bar{Q}}_{0}$ on self-oscillation. (**a**) Amplitude and $\varepsilon_{0}$. (**b**) Frequency and $\varepsilon_{0}$. The illustration indicates that the values of amplitude and frequency are independent of the initial electrothermal intensity.

Figure 9 depicts the curves of amplitude, frequency, and $\varepsilon_{0}$ as functions of the initial electrothermal intensity ${\bar{Q}}_{0}$, and the results indicate that variations in ${\bar{Q}}_{0}$ have no impact on any of the three. The values of the other parameters are $\bar{\alpha }=0.35$, ${\bar{C}}_{1}=0.01$, $\bar{\tau }=0.01$, $\bar{\beta }=5$, $\bar{j}=0.2$, ${\bar{C}}_{2}=0.2$, ${\bar{L}}_{a}=1.9$, and ${\bar{L}}_{s}=0.8$. From Figure 9a, when $\varepsilon_{0}>0$, the LCE self-oscillator is in a self-oscillating phase. The values of amplitude and $\varepsilon_{0}$ remain steady and fixed at certain values as ${\bar{Q}}_{0}$ increases. Similarly, from Figure 9b, it can be observed that the frequency of the self-oscillation process remains unchanged regardless of alterations in ${\bar{Q}}_{0}$. The initial electrothermal intensity directly affects the amount of electrothermal energy absorbed by the LCE fiber. A higher initial electrothermal intensity results in a greater strain upper limit during the self-oscillation process, while the strain lower limit also increases. Consequently, this results in an enhancement in both the upper and lower displacement limits of the mass, confirming that the amplitude remains unchanged, as shown in the diagram. This finding reveals that the initial electrothermal intensity does not influence the motion phase of the LCE self-oscillator.

**Figure 10:**
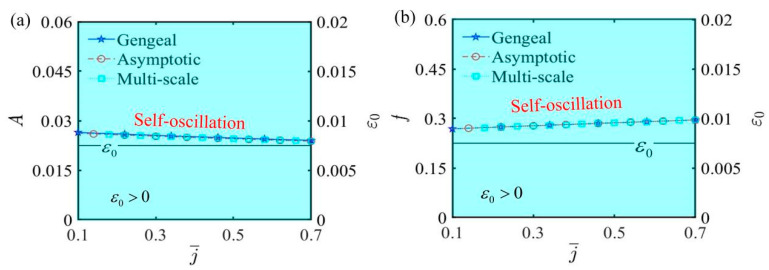
The influence of the spring elastic coefficient $\bar{j}$ on self-oscillation. (**a**) Amplitude and $\varepsilon_{0}$. (**b**) Frequency and $\varepsilon_{0}$. Increasing the spring elastic coefficient only results in a very slight decrease in amplitude and a minor increase in frequency.

Figure 10 depicts the curves of amplitude, frequency, and $\varepsilon_{0}$ as functions of the spring elastic coefficient $\bar{j}$, and other parameters include $\bar{\alpha }=0.35$, ${\bar{C}}_{1}=0.01$, $\bar{\tau }=0.01$, $\bar{\beta }=5$, ${\bar{Q}}_{0}=0.8$, ${\bar{C}}_{2}=0.2$, ${\bar{L}}_{a}=1.9$, and ${\bar{L}}_{s}=0.8$. Due to small deformations, the effect of the spring elastic coefficient on self-oscillation is not prominently highlighted in the function curve graphs. Actually, from Figure 10a,b, we can conclude that when $\varepsilon_{0}$ remains a constant value greater than zero, the self-oscillation amplitude experiences a slight increase, and the frequency correspondingly diminishes as the spring elastic coefficient increases. This phenomenon can be explained by the characteristic of the spring: a higher elastic coefficient increases the rigidity of the spring, thereby constraining the displacement response of the system during self-oscillation, which suppresses the increase in amplitude. On the other hand, increasing the spring elastic coefficient means that the spring can recover more quickly from the strain deformation caused by the force acting on the LCE fiber, leading to a shorter time experienced during one cycle of motion, ultimately reflected in the increased frequency shown in the graph. Therefore, selecting an appropriate spring can effectively enhance the realization and application of self-oscillation.

**Figure 11:**
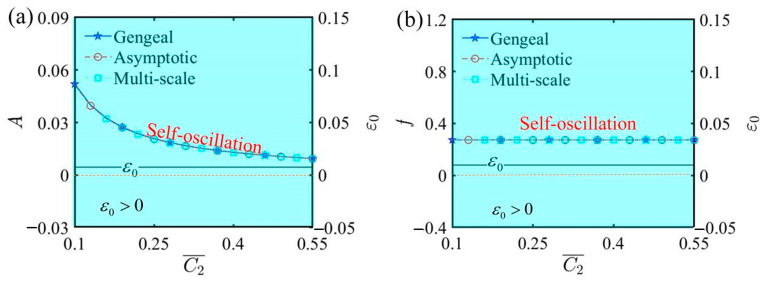
The influence of the second damping coefficient ${\bar{C}}_{2}$ on self-oscillation. (**a**) Amplitude and $\varepsilon_{0}$. (**b**) Frequency and $\varepsilon_{0}$. The second damping coefficient exerts a considerable influence on the amplitude of self-oscillation, while it has almost no effect on the frequency.

Figure 11 illustrates the relationship curves among amplitude, frequency, and $\varepsilon_{0}$ as they vary with the second damping coefficient ${\bar{C}}_{2}$. The additional parameters are as follows: $\bar{\alpha }=0.35$, ${\bar{C}}_{1}=0.01$, $\bar{\tau }=0.01$, $\bar{\beta }=5$, ${\bar{Q}}_{0}=0.8$, $\bar{j}=0.2$, ${\bar{L}}_{a}=1.9$, and ${\bar{L}}_{s}=0.8$. The Hurwitz criterion indicates that the value of $\varepsilon_{0}$ remains positive, signifying no bifurcation in amplitude or frequency. Consequently, the LCE self-oscillator consistently remains in a self-oscillating phase. From Figure 11, it is also clear that whether ${\bar{C}}_{2}$ increases or decreases, the self-oscillating phase remains unchanged. Additionally, an increase in the second damping coefficient produces a nonlinear diminution in amplitude, according to Figure 11a. Changes in ${\bar{C}}_{2}$ affect the amplitude of self-oscillation because it introduces an additional nonlinear damping term, increasing energy loss; however, it does not alter the frequency. Therefore, by effectively controlling the second damping coefficient and utilizing the characteristic of varying the amplitude without changing the frequency, specific functions of the LCE self-oscillator can be achieved.

**Figure 12:**
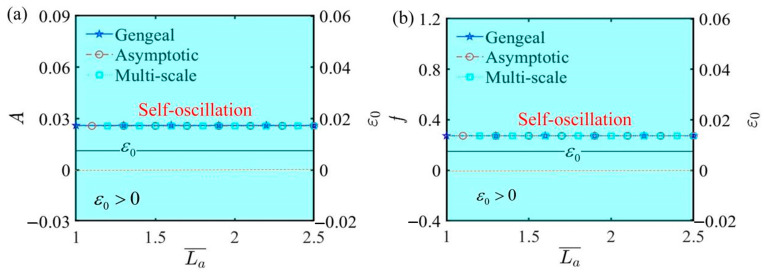
The influence of fixed end length ${\bar{L}}_{a}$ on self-oscillation. (**a**) Amplitude and $\varepsilon_{0}$. (**b**) Frequency and $\varepsilon_{0}$. The fixed end length exerts negligible influence on the amplitude and frequency of self-oscillation.

Figure 12 plots the effect of fixed end length ${\bar{L}}_{a}$ on self-oscillation under the parameters $\bar{\alpha }=0.35$, ${\bar{C}}_{1}=0.01$, $\bar{\tau }=0.01$, $\bar{\beta }=5$, ${\bar{Q}}_{0}=0.8$, $\bar{j}=0.2$, ${\bar{C}}_{2}=0.2$, and ${\bar{L}}_{s}=0.8$. In accordance with the Hurwitz criterion, the value of $\varepsilon_{0}$ invariably remains positive, indicating the lack of any bifurcation in both amplitude and frequency. Consequently, the LCE self-oscillator maintains a steady motion in the self-oscillating phase. Figure 12 also demonstrates that elongating or shortening the fixed end length does not affect the change in the motion phase. The results from the curves indicate that selecting a reasonable fixed end length for the experimental device allows the LCE self-oscillator to complete a full cycle of self-oscillation.

**Figure 13:**
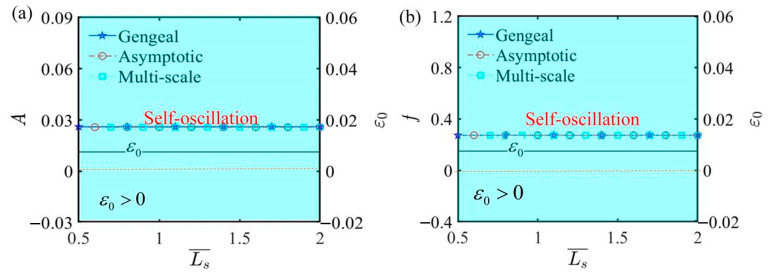
The influence of spring original length ${\bar{L}}_{s}$ on self-oscillation. (**a**) Amplitude and $\varepsilon_{0}$. (**b**) Frequency and $\varepsilon_{0}$. The illustration shows that ${\bar{L}}_{s}$ exerts no influence on the amplitude and frequency of self-oscillation.

Figure 13 plots the effect of spring original length ${\bar{L}}_{s}$ on self-oscillation under the parameters $\bar{\alpha }=0.35$, ${\bar{C}}_{1}=0.01$, $\bar{\tau }=0.01$, $\bar{\beta }=5$, ${\bar{Q}}_{0}=0.8$, $\bar{j}=0.2$, ${\bar{C}}_{2}=0.2$, and ${\bar{L}}_{a}=1.9$. Within these combinations of parameters, $\varepsilon_{0}$ is determined to be a fixed value, ensuring that the self-oscillating phase remains unchanged. From the analysis of the parameters ${\bar{L}}_{a}$ and ${\bar{L}}_{s}$, it is clear that they leave the bifurcation points unchanged, further validating the accuracy of the Hurwitz criterion. This result also demonstrates that the amplitude and frequency of LCE self-oscillation depend on the internal material properties and molecular structure, rather than by external dimensional information.

From the analysis of the above influential and non-influential parameters in various diagrams, we can reasonably assert that the numerical solutions obtained from both the governing and asymptotic equations, along with the multi-scale analytical solutions, consistently align with each other. In conjunction with Equation (39), the parameters $\bar{j}$, $\bar{\alpha }$, and $\bar{\beta }$ exert a monotonic effect on the frequency, preventing the attainment of a maximum value, while the other parameters have no effect on the frequency. This further corroborates the influence mechanism of each parameter on the frequency as observed in the figures.

## 7. Conclusions

To overcome the dependence of feedback mechanisms in conventional light-fueled self-oscillating systems on spatially distributed light and intricately designed structures, this paper preliminarily establishes an electrically driven LCE self-oscillator based on the rheostat feedback mechanism. In the established electrically driven nonlinear model, the numerical results indicate that the LCE self-oscillator can exhibit two phases under different parameter combinations: stationary and periodically oscillating in a horizontal direction. By utilizing the deformation of the LCE to couple the movement of the rheostat slider for current adjustment, the LCE fiber dynamically absorbs electrothermal energy, resulting in periodic stretching and contraction. This behavior is maintained by continuously compensating for the damping dissipation of the mass with the supplied electrical energy.

Additionally, by applying the Hurwitz criterion, the parameters were classified into those that influence bifurcation points and those that do not, enabling a detailed analysis of the amplitude and frequency of the LCE self-oscillator. Numerical methods and the multi-scale method were employed to solve for amplitude and frequency, confirming the consistency of the results. Notably, the analysis of the influential parameters identified critical values that trigger self-oscillation, providing deeper insights into the underlying mechanisms of motion. By appropriately adjusting the system parameters, the performance of self-oscillation can be optimized, which is of significant importance for the development and exploration of self-oscillating systems.

The present model of electrically driven LCE self-oscillators utilizing a rheostat feedback mechanism operates under the assumption that the elasticity coefficient is independent of temperature variations. Additionally, it posits a direct proportionality between temperature and the electrically driven strain. Although such an approach streamlines the analysis and offers qualitative understanding, it might not fully account for the intricate nonlinear interplay among these factors, particularly when pursuing precise quantitative outcomes. To enhance predictive precision, future research should investigate two key aspects: the temperature-dependent nature of the elasticity coefficient and the impact of the nonlinear relationship between temperature and electrically driven strain. Notwithstanding these constraints, the self-oscillator stands out for its beneficial traits, such as simplified design, adjustable dimensions, and rapid response. The findings offer a valuable understanding of LCE self-oscillation behavior and suggest numerous application concepts for design in fields such as soft robots, sensors, and energy harvesters.

## Data Availability

Data will be made available upon request.
